# Cone-Beam Computed Tomography Assessment of Lower Facial Asymmetry in Unilateral Cleft Lip and Palate and Non-Cleft Patients with Class III Skeletal Relationship

**DOI:** 10.1371/journal.pone.0130235

**Published:** 2015-08-03

**Authors:** Yifan Lin, Gui Chen, Zhen Fu, Lian Ma, Weiran Li

**Affiliations:** 1 Department of Orthodontics, Peking University School and Hospital of Stomatology, Beijing, China; 2 Department of Oral Maxillofacial Surgery, Peking University School and Hospital of Stomatology, Beijing, China; Medical University of South Carolina, UNITED STATES

## Abstract

**Introduction:**

To evaluate, using cone-beam computed tomography (CBCT), both the condylar-fossa relationships and the mandibular and condylar asymmetries between unilateral cleft lip and palate (UCLP) patients and non-cleft patients with class III skeletal relationship, and to investigate the factors of asymmetry contributing to chin deviation.

**Methods:**

The UCLP and non-cleft groups consisted of 30 and 40 subjects, respectively, in mixed dentition with class III skeletal relationships. Condylar-fossa relationships and the dimensional and positional asymmetries of the condyles and mandibles were examined using CBCT. Intra-group differences were compared between two sides in both groups using a paired t-test. Furthermore, correlations between each measurement and chin deviation were assessed.

**Results:**

It was observed that 90% of UCLP and 67.5% of non-cleft subjects had both condyles centered, and no significant asymmetry was found. The axial angle and the condylar center distances to the midsagittal plane were significantly greater on the cleft side than on the non-cleft side (P=0.001 and P=0.028, respectively) and were positively correlated with chin deviation in the UCLP group. Except for a larger gonial angle on the cleft side, the two groups presented with consistent asymmetries showing shorter mandibular bodies and total mandibular lengths on the cleft (deviated) side. The average chin deviation was 1.63 mm to the cleft side, and the average absolute chin deviation was significantly greater in the UCLP group than in the non-cleft group (P=0.037).

**Conclusion:**

Compared with non-cleft subjects with similar class III skeletal relationships, the subjects with UCLP showed more severe lower facial asymmetry. The subjects with UCLP presented with more asymmetrical positions and rotations of the condyles on axial slices, which were positively correlated with chin deviation.

## Introduction

Cleft lip and palate (CLP) is a congenital facial anomaly characterized by underdevelopment of the maxilla due to surgical repair, scar contracture and/or congenital growth deficiency. Regarding the anteroposterior dimension, CLP subjects generally present with anterior crossbite and show a tendency toward class III malocclusion [1]. In Ross’s report based on cephalograms from 15 centers worldwide, the incidence rate of maxillary retrusion requiring orthognathic surgery was approximately 25% [2–5]. Similarly, Mars et al evaluated dental models from six centers and reported an indication for orthognathic surgery for 10–50% of patients [6]. According to other reports, the potential for maxillary growth in unilateral CLP (UCLP) subjects is similar to that in non-cleft subjects, and the deficiency of maxillary growth may result from cleft repair surgery [7–9].

Considering facial asymmetry in the transverse dimension, subjects with CLP, particularly those with UCLP, often present with various degrees of facial asymmetry. Several studies have reported asymmetrical and distorted features of the naso-maxillary complex in UCLP patients with consistent results [10–14]. However, regarding the mandible, various studies have suggested different ideas [15–17].

By examining panoramic radiographs, Kurt et al reported increased gonial angles on the cleft side in subjects with UCLP [15]. According to Laspos et al, subjects with UCLP did not present with significant mandibular asymmetry, whereas the asymmetry of the lower facial skeleton was attributed to possible cranial-base/temporal-region anomalies, and the asymmetry increased with growth [16]. Abad-Santamaría et al reported no significant differences in mandibular asymmetry among UCLP, unilateral posterior crossbite, and normal occlusion subjects on panoramic radiographs [17].

The inconsistencies in the above-mentioned studies were most likely due to (1) the mandible not being directly affected by the cleft and (2) technical difficulties associated with the multifactorial etiology of lower facial asymmetry. Specifically, these developmental factors include true mandibular asymmetry, mandibular positional adaptation to asymmetrical fossa of temporomandibular joint (TMJ), and functional adaptation to dentoalveolar and occlusal disharmonies. In addition, abnormal muscular function and scar contracture caused by surgeries might have resulted in adaptive and compensational changes in the condylar growth centers.

Conventional 2-D imaging, including panoramic, posteroanterior and oblique cephalometric radiographs, has suffered from magnification and distortion errors, and its reliability has been limited, particularly in the evaluation of facial asymmetry, due to the superimposition of important structures and difficulties in landmark identification [18–20]. Previous studies have suggested the high reliability and accuracy of cone-beam computed tomography (CBCT) imaging in the evaluation of craniofacial structures [21–24].

In three-dimensional imaging evaluations, Veli et al reported that subjects with UCLP had symmetrical mandibles except for a longer coronoid unit on the cleft side [25]. Celikoglu et al reported lower ramal height and shorter ramal plus condylar height on the cleft side in subjects with UCLP, in addition to an increased asymmetry index [26]. Kim et al investigated the relationships between chin deviation and the position and morphology of the mandibles in 28 UCLP adults using CBCT imaging [27] and found that the chin deviated to cleft side by 1.59 mm, the vertical positions of temporomandibular fossa and condyle were lower, and mandibular body length was shorter on the cleft side.

Until now, few studies have compared condylar-fossa relationships and bilateral mandibular asymmetries between UCLP and non-cleft subjects or investigated the factors contributing to chin deviation. Additionally, most previous studies enrolled non-cleft subjects with normal occlusion as the control groups, although it is known that subjects with CLP often present with varying degrees of class III malocclusion and skeletal discrepancies. Compared with non-cleft subjects with normal occlusion, patients with CLP have more severe sagittal discrepancies and transverse asymmetries [28–30].

Whether a unilateral cleft would have an impact on the morphology and position of the condyles and the mandibles and thus affect the lower facial symmetry of skeletal class III UCLP subjects, compared with class III non-cleft subjects, is unknown.

Therefore, the current study aimed using CBCT imaging: (1) to evaluate the condylar-fossa relationships and dimensional and positional asymmetries of condyles between the two sides, (2) to evaluate mandibular dimensional asymmetries, and (3) to investigate the relationship between mandibular asymmetry and chin deviation in UCLP and non-cleft subjects with class III skeletal relationship.

## Materials and methods

This research was approved by the Ethics Committee of the Peking University School of Stomatology, China (PKUSSIRB-2012049). All of the participants and their parents provided written informed consent, and all of the clinical investigations were conducted according to the principles of the Declaration of Helsinki.

### 2.1 Subjects

All subjects were Chinese residents of Northern Chinese origin. The subjects were selected according to the following criteria. The inclusion criteria for the UCLP group were (1) operated non-syndromic UCLP; (2) class III skeletal relationship with maxillary hypoplasia; (3) concave profile with an ANB angle of >-4 degree and <1 degree, and anterior crossbite; (4) mixed dentition and cervical vertebral maturation stage between CVMS1 and CVMS3 [31]; and (5) no previous orthodontic treatment.

Included in the UCLP group were 30 children (20 boys, 10 girls) between the ages of 7.5 and 12.0 years old (mean 10.31 years, SD = 1.23). Of the subjects with UCLP, 23 (76.67%) had a cleft on the left side, and 7 (23.33%) had a cleft on the right side.

All of the subjects with UCLP underwent cheiloplasty prior to turning 1 year old, palatoplasty prior to turning 3 years old, and alveolar bone grafting surgery at least 3 months prior to starting this study. All of the surgeries were performed at the Cleft Lip and Palate Treatment Center, Peking University School of Stomatology, China.

The inclusion criteria for the non-cleft group were similar to those for the UCLP group. The subjects were selected from the Department of Orthodontics, Peking University School of Stomatology, China. A total of 40 children (16 boys, 24 girls) between the ages of 7.6 and 12.3 years old (mean 9.70 years, SD = 1.32) were included in the non-cleft group.

Clinical examination of the TMJ was performed. No clinical signs or symptoms of temporomandibular dysfunction, including pain, joint sounds, and abnormal joint movement, were found in the subjects in either group.

### 2.2 Methods

Evaluations were performed using CBCT, and the scans were obtained using a CBCT machine (DCT Pro; Vatech & EWOO Group, South Korea). The patient was seated in a chair with a natural head position oriented by an experienced clinician, in maximum intercuspal occlusion with a relaxed tongue and passive lips maintained. All of the scans were obtained using the following protocol: field of view, 200 × 190 mm^2^; 90 Kvp; 144 mA; scan time, 24 s; voxel size, 0.4 mm^3^. The CBCT data were converted into the Digital Imaging and Communications in Medicine (DICOM) file format. Digitization and measurement were performed using Dolphin Imaging Software (version 11.7, Dolphin Imaging & Management Solutions, Chatsworth, CA, USA).

For convenience of comparison, in the UCLP group, the 7 UCLP patients with a right-sided cleft were all set to the opposite (left) side, which was easily accomplished by changing the x coordinates.

In the non-cleft group, to clarify the asymmetry between sides and to avoid it being neutralized by deviation to the left and right sides, the subjects were classified and compared according to the deviated side and the non-deviated side. Additionally, the deviated side of the non-cleft group was set to the left, and the non-deviated side was set to the right. Therefore, the values of chin deviation in the non-cleft group were all positive.

### 2.3 Reference planes

**Fig 1:**
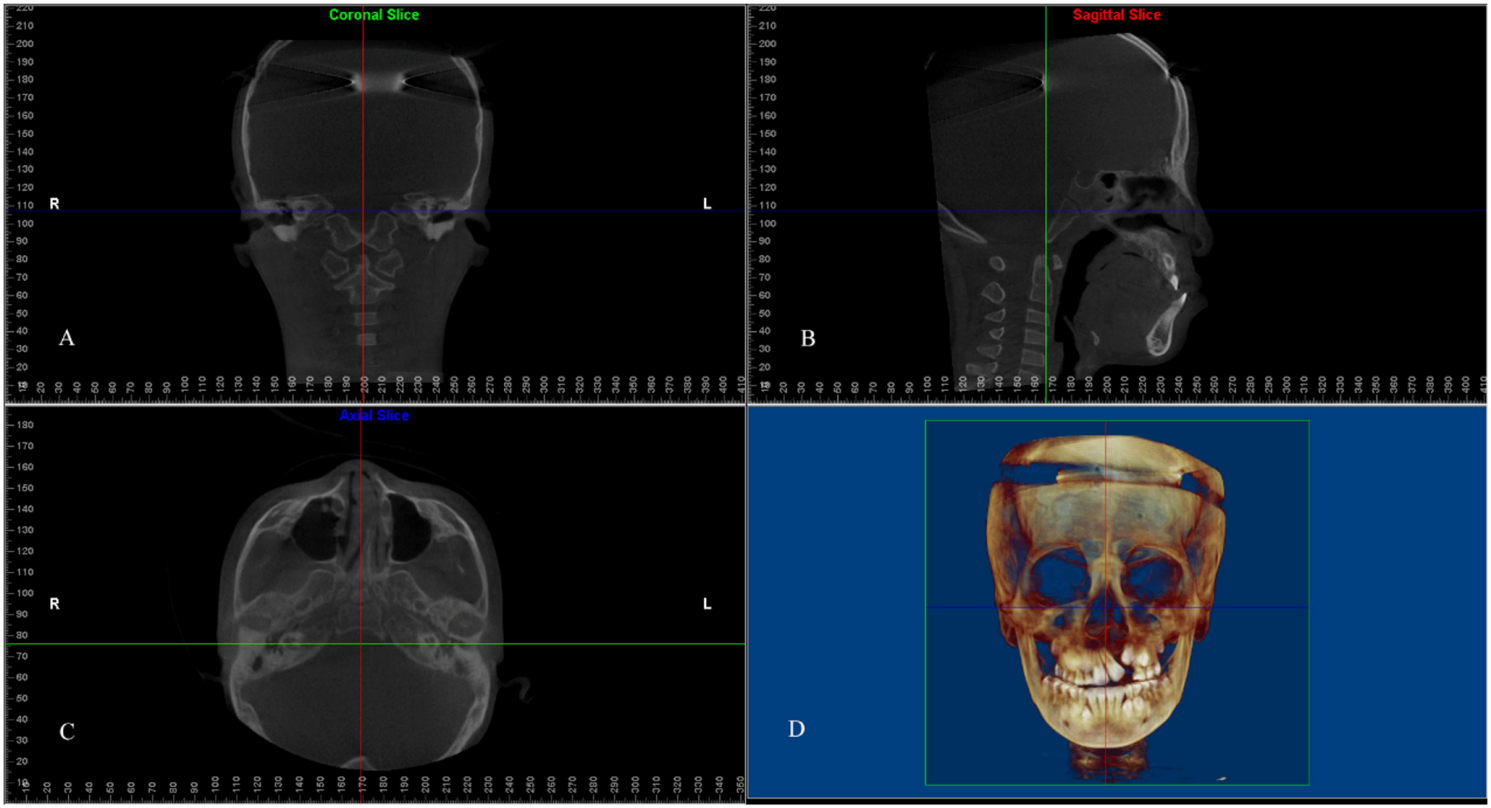
Head orientation and reference planes. (A) Coronal plane; (B) Midsagittal plane (MSP); (C) Frankfort horizontal (FH) plane; (D) Three-dimensional rendered image.

Descriptions of landmarks and reference planes are shown in Table 1. The CBCT images were carefully oriented in three dimensions (Fig 1), according to the protocol suggested by Damstra [32]: (1) the Frankfort horizontal (FH) plane passed through the bilateral poria and orbitale on the unaffected side of the UCLP subjects (for non-cleft subjects, the right side was used) and was parallel to the ground; (2) the midsagittal plane (MSP) passed through the sella (S) and basion (Ba) points and was perpendicular to the FH plane; (3) the coronal plane passed through the Ba point and was perpendicular to the sagittal and FH planes.

**Table 1:** Descriptions of landmarks.

Landmark	Abbreviation	Description
**Unilateral**		
Sella	S	The center of the sella turcica
Basion	Ba	The most anterior margin of the foramen magnum
Menton	Me	The most inferior midpoint on the mandibular symphysis
**Bilateral**		
Orbitale	Or	The lowest point in the inferior margin of the orbit
Porion	Po	The most superior point on the roof of the external auditory meatus
Gonion	Go	The point on the curvature of the angle of the mandible located by bisecting the angle formed by lines tangent to the posterior ramus and the inferior border of the mandible
Center of condyle	Co_cen_	The bisecting point of the ML and AP axes of the condyle on the axial plane
Condylion superius	Co_sup_	The most superior point of the condyle head
Condylion posterius	Co_post_	The most posterior point of the condyle head
**Reference planes**		
Frankfort plane	FH plane	Plane connecting bilateral Po and Or on the non-cleft side (for non-cleft subjects, on the non-deviated side), oriented horizontally parallel to the floor
Midsagittal plane	MSP	Passing through Ba and S, perpendicular to the FH plane
Coronal plane		Passing through Ba, perpendicular to the FH and MSP planes
**Reference planes for TMJ**		
Axial plane		Plane parallel to the FH plane showing maximal cross-sectional area of the condyle
Sagittal plane		Plane passing through the AP axis of the condyle and perpendicular to the FH plane
Coronal plane		Plane passing through the ML axis of the condyle and perpendicular to the FH plane

**Fig 2:**
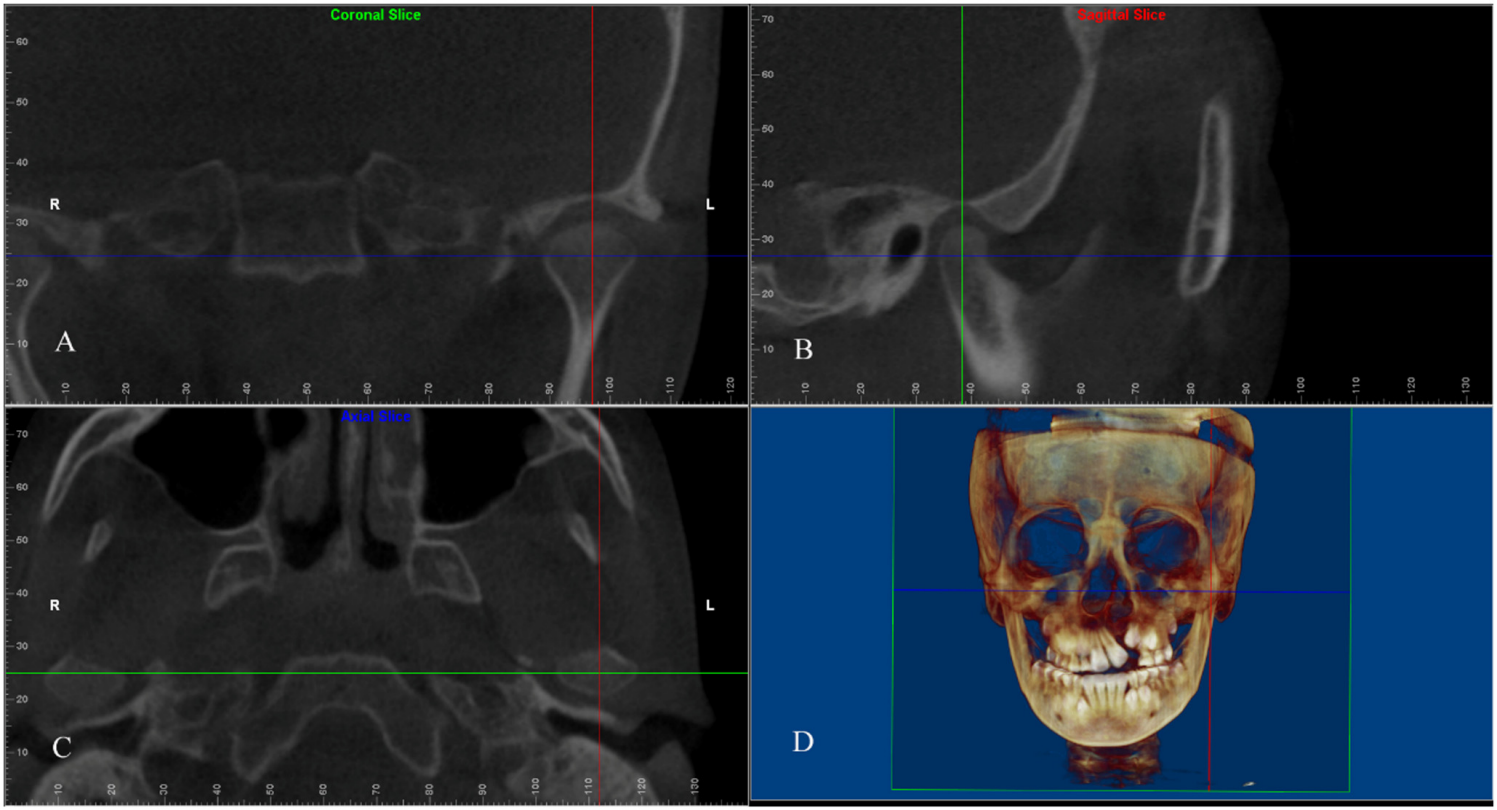
Reference planes of the temporomandibular joint (TMJ). (A) Coronal plane of TMJ on the cleft side; (B) Sagittal plane of TMJ on the cleft side; (C) Axial plane of TMJ on the cleft side; (D) Three-dimensional rendered image.

Prior to obtaining condylar measurements, a local coordinate system of the TMJ was constructed (Fig 2). The axial plane of the TMJ was defined as a plane parallel to the FH plane showing maximum cross-sectional area of the condyle. The mediolateral (ML) axis of the condyle was represented by the longest ML diameter of the condyle on the axial plane. The coronal plane of the TMJ passed through the ML axis perpendicular to the axial plane. The anteroposterior (AP) axis bisected and was perpendicular to the ML axis on the axial plane and represented the AP diameter of the condyle. The sagittal plane passed through the AP axis of the condyle perpendicular to the axial and coronal planes of the TMJ.

### 2.4 Condylar-fossa relationships and condylar concentricity on the sagittal plane

**Fig 3:**
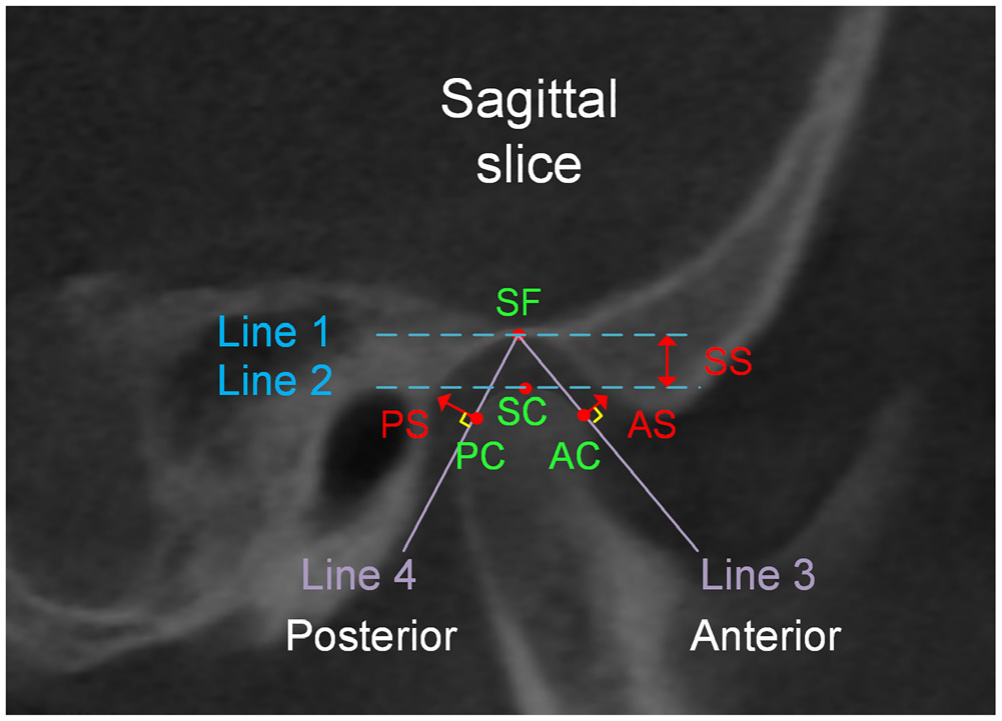
Landmarks and measurements of condylar-fossa relationship on a sagittal slice. SF, the most superior aspect of the temporomandibular fossa; SC, the most superior aspect of the condyle; PC, the posterior tangent point of the condyle; AC, the anterior tangent point of the condyle; Line 1, tangent to SF and parallel to the FH plane; Line 2, tangent to SC and parallel to line 1; Line 3, starting from SF and tangent to the most anterior aspect of the condyle; Line 4, starting from SF and tangent to the most posterior aspect of the condyle; PS, posterior joint space; SS, superior joint space; AS, anterior joint space.

Assessment of condylar-fossa relationships was performed by measuring the joint space between the temporomandibular fossa and condyle using the sagittal view of the TMJ (Fig 3). Digitization and measurements were performed using Digimizer software (version 4.2, MedCalc software, Mariakerke, Belgium). Landmarks and variables are presented in Tables 1 and 2. The following joint space measurements were assessed according to the method proposed by Kamelchuk [33] (Table 2): (1) anterior space; (2) superior space; (3) posterior space.

**Table 2:** Descriptions of measurements.

Measurement	Description
**Condylar-fossa relationship and condylar concentricity in the sagittal plane**	
Superior joint space (SS)	Distance between the most superior aspect of the temporomandibular fossa and the most superior aspect of the condyle
Anterior joint space (AS)	Distance between the anterior aspect of the temporomandibular fossa and the anterior tangent point of the condyle
Posterior joint space (PS)	Distance between the posterior aspect of the temporomandibular fossa and the posterior tangent point of the condyle
Condylar concentricity	${\text{log}}_{e}\frac{P}{A}$
**Dimension and position of condyle on axial plane**	
AP diameter of condyle	Length of the AP axis of the condyle
ML diameter of condyle	Length of the ML axis of the condyle
Axial angle of condylar process	Angle between the ML axis of the condyle and the MSP
Condylar center distance to MSP	Distance from the geometric center of the condyle to the MSP
AP difference of condylar process	AP distance between the geometric centers of the condyles on the two sides
Vertical difference of condylar process	Vertical distance between the geometric centers of the condyles on the two sides
**Mandibular dimension and chin deviation**	
Ramal height	Distance between Co_post_ and Go
Mandibular body length	Distance between Go and Me
Total mandibular length	Distance between Co_sup_ and Me
Gonial angle	Angle between the Me-Go and Co_post_-Go vectors
Chin deviation	Perpendicular distance from Me to the MSP

P, posterior joint space; A, anterior joint space; AP, anteroposterior; ML, mediolateral.

The quantitative method for evaluating condylar concentricity was expressed as ${\text{log}}_{e}\frac{P}{A}$, where P is posterior joint space and A is anterior joint space, as proposed by Pullinger [34]. Condylar concentricity was defined as a range of ±0.25 on the ${\text{log}}_{e}\frac{P}{A}$ scale; thus, the condyle was considered to be anteriorly positioned when ${\text{log}}_{e}\frac{P}{A}$ was greater than 0.25 and to be posteriorly positioned when the result was less than -0.25.

### 2.5 Dimension and position of the condyles on the axial plane

**Fig 4:**
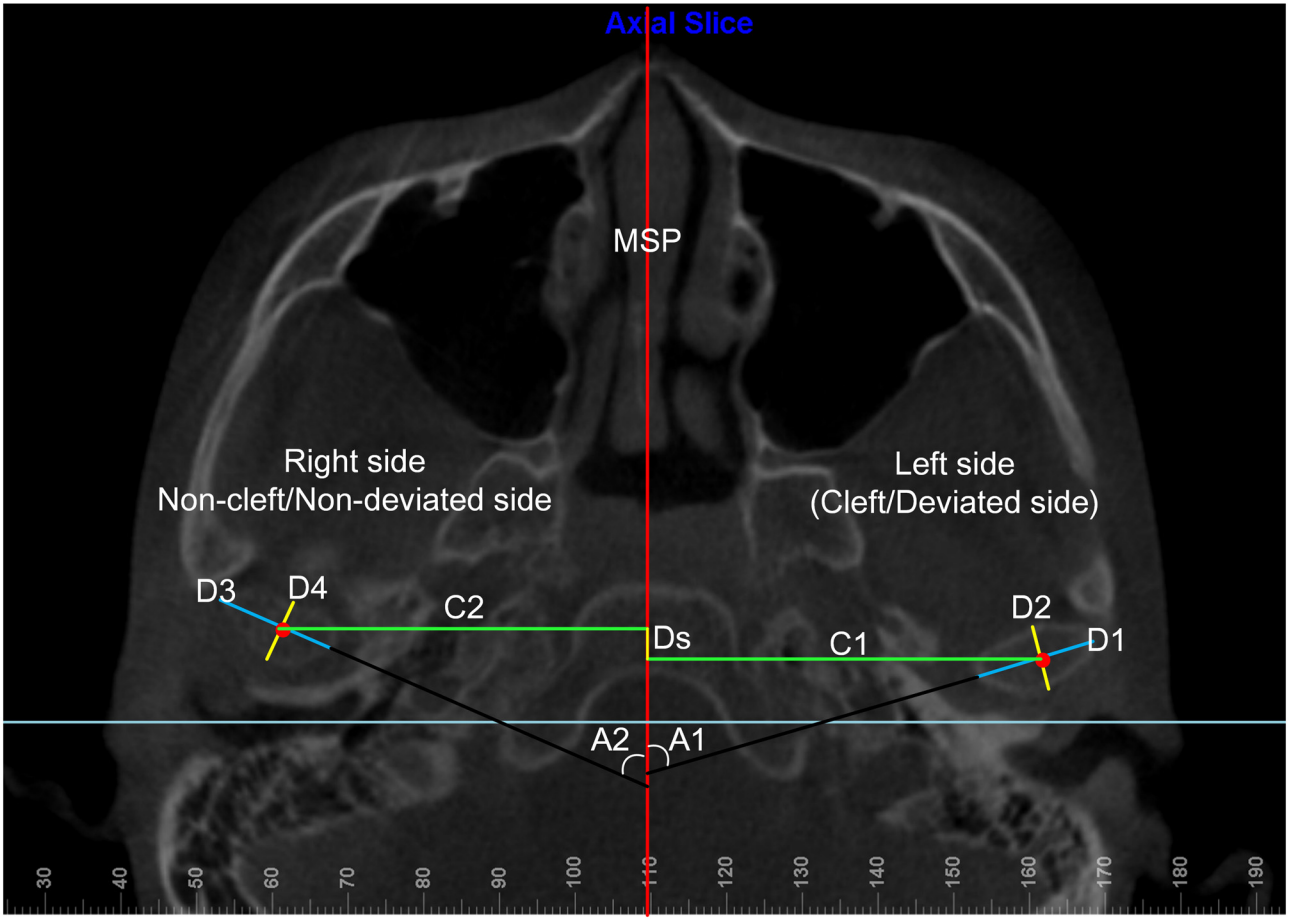
Dimensions and positions of condyles on an axial slice. D1 and D3, the mediolateral (ML) diameters of condyles; D2 and D4, the anteroposterior (AP) diameters of condyles; Ds, the sagittal difference between the geometric centers of condyles on two sides; C1 and C2, the distances between the center of condyles and the MSP; A1 and A2, the angles between the ML axis of condyles and the MSP.

Axial slices were used to assess the dimensional and positional asymmetry of the bilateral condyles. The following measurements of the condyles on the cleft and non-cleft sides (for non-cleft patients, the deviated and non-deviated sides were used) were independently assessed on axial planes (Table 2 and Fig 4): (1) ML diameter; (2) AP diameter; (3) axial angle between the ML axis of the condyle and the MSP; (4) distance from the center of condyle to the MSP; (5) AP difference between the centers of condyles on the two sides; (6) vertical difference between the centers of condyles on the two sides.

In the UCLP group, the center of the condyle on the non-cleft side was set as point 0. The variations on the cleft sides were measured from this point. The centers of condyles on the cleft sides, situated anteriorly and superiorly to point 0, were considered positive, and those situated posteriorly and inferiorly to point 0 were considered negative. Similarly, in the non-cleft group, the center of the condyle on the non-deviated side was set as point 0. The centers of condyles on the deviated sides situated anteriorly and superiorly to point 0 were considered positive, and those situated posteriorly and inferiorly to point 0 were considered negative.

### 2.6 Mandibular dimension and chin deviation

**Fig 5:**
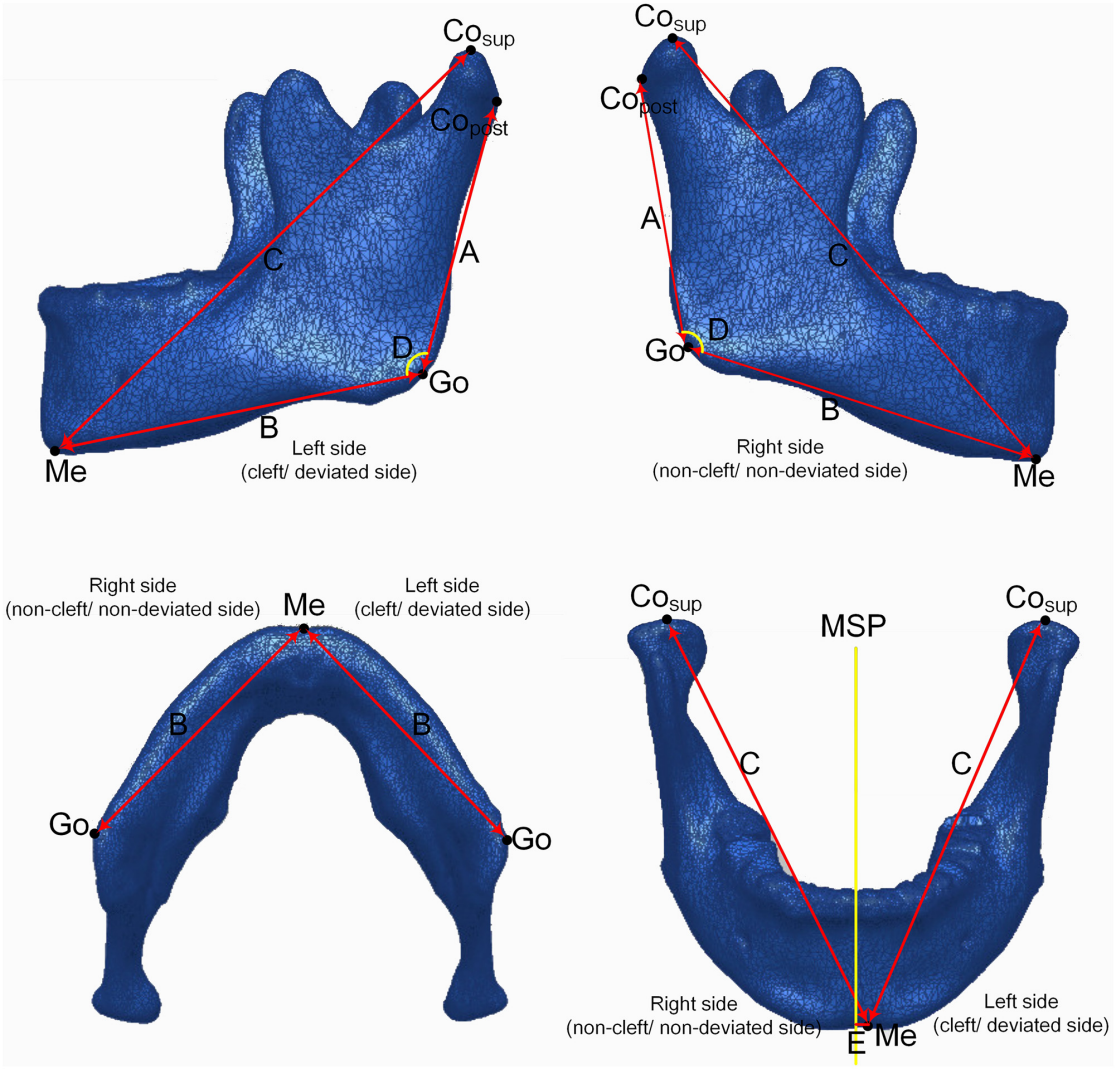
Mandibular measurements and chin deviation. (A) Ramal height; (B) Mandibular body length; (C) Total mandibular length; (D) Gonial angle; (E) Chin deviation.

Mandibular dimension and chin deviation were assessed using the following measurements (Table 2 and Fig 5): (1) ramal height; (2) mandibular body length; (3) total mandibular length; (4) gonial angle; (5) chin deviation. Chin deviation to the cleft (deviated) side was considered positive, whereas deviation to the non-cleft side was considered negative.

### 2.7 Statistical analysis

To evaluate intra-observer reliability, 10 CBCT images were randomly selected from the two groups and were re-measured 2 weeks later by the same investigator. Random error was calculated using Dahlberg’s formula, $D=\sqrt{\frac{\sum d^{2}}{2n}}$, where d is the difference between the first and second measurements, and n is the sample size that was re-measured [35]. The results of the error for linear and angular measurements were within 1 mm and 1 degree, respectively. To evaluate inter-observer reliability, 10 randomly chosen CBCT images were measured by another investigator. The intraclass correlation coefficient (ICC) ranged from 0.80 to 0.99, indicating a high level of reliability.

Student’s paired t test was performed to evaluate the differences in each measurement between the cleft and non-cleft sides in the UCLP subjects and between the deviated and non-deviated sides in the non-cleft subjects. Pearson correlation analysis was performed to investigate the correlations between chin deviation and each variable. Values of P<0.05 were considered statistically significant. All of the statistical analyses in the current study were performed using the SPSS statistical software package, version 13.0 (SPSS, Chicago, IL, USA).

## Results

Comparisons of the measurements between the cleft and non-cleft sides in the UCLP group are shown in Table 3.

**Table 3:** Comparison of the measurements between the cleft and non-cleft side (paired t test), and correlation with chin deviation (Pearson correlation analysis) in the UCLP group.

Measurement	Cleft side		Non-cleft side		Difference			Correlation with chin deviation	
	Mean	SD	Mean	SD	Mean	SD	P	r	P
**Condylar-fossa relationship and condylar concentricity on sagittal plane**									
SS (mm)	2.54	0.60	2.43	0.48	0.11	0.59	0.323	-0.168	0.374
AS (mm)	1.73	0.23	1.73	0.19	0.00	0.29	0.965	-0.255	0.173
PS (mm)	2.08	0.25	2.02	0.23	0.05	0.33	0.408	-0.311	0.094
Concentricity	0.18	0.06	0.16	0.05	0.02	0.07	0.069	-0.145	0.446
**Dimension and position of condyle on axial plane**									
ML diameter (mm)	16.07	1.81	16.18	1.65	-0.11	0.83	0.474	-0.292	0.118
AP diameter (mm)	7.70	0.77	7.85	0.63	-0.15	0.55	0.147	-0.285	0.127
Axial angle (degree)	72.45	5.84	69.46	7.03	2.98	3.75	0.001*	0.358	0.052
Center distance to MSP (mm)	50.12	2.55	49.14	2.90	0.97	2.31	0.028*	0.365	0.047*
AP difference of condyle (mm)	-0.44	2.45	0.00	0.00	-0.44	2.45	0.329	-0.315	0.090
Vertical difference of condyle (mm)	0.53	1.88	0.00	0.00	0.53	1.88	0.135	0.195	0.301
**Mandibular dimension**									
Ramal height (mm)	46.59	4.00	46.99	3.47	-0.41	1.99	0.273	-0.386	0.035*
Mandibular body length (mm)	71.69	4.34	72.50	4.51	-0.80	1.91	0.028*	-0.414	0.023*
Total mandibular length (mm)	107.06	5.25	108.07	5.45	-1.02	1.66	0.002*	-0.427	0.018*
Gonial angle (degree)	127.16	3.45	126.06	4.05	1.09	2.31	0.015*	0.200	0.289

SS, superior joint space; AS, anterior joint space; PS, posterior joint space; ML, mediolateral; AP, anteroposterior.

* P<0.05.

In the UCLP group, no significant differences were found in superior, anterior or posterior joint space between the cleft and non-cleft sides. The ${\text{log}}_{e}\frac{P}{A}$ value was 0.18 for the cleft side and 0.16 for the non-cleft side, with no significant difference between the two sides (P = 0.069).

Regarding the dimensions and positions of the condylar processes, no statistically significant differences between the cleft and non-cleft sides were found in the AP and ML dimensions. The average distances from the geometric center of the condylar process to the MSP were 50.12 mm on the cleft side and 49.14 mm on the non-cleft side (P = 0.028). The average value of the angle between the ML axis of the condyle and the MSP on the cleft side was 72.45 degrees; however, the angle on the non-cleft side was significantly lower, at 69.46 degrees (P = 0.001). The average AP and vertical positional differences in the condylar processes were not significant between the cleft and non-cleft sides.

In evaluating mandibular dimension, the mandibular body and the total mandibular length were significantly shorter on the cleft side than on the non-cleft side (P = 0.028 and P = 0.002, respectively). The gonial angle was significantly larger on the cleft side than on the non-cleft side (P = 0.015).

Comparisons of the measurements between the deviated and non-deviated sides in the non-cleft group are shown in Table 4.

**Table 4:** Comparison of the measurements between the deviated and non-deviated side (paired t test), and correlation with chin deviation (Pearson correlation analysis) in the non-cleft group.

Measurement	Deviated side		Non-deviated side		Difference			Correlation with chin deviation	
	Mean	SD	Mean	SD	Mean	SD	P	r	P
**Condylar-fossa relationship and condylar concentricity on sagittal plane**									
SS (mm)	2.18	0.54	2.15	0.56	0.03	0.55	0.704	-0.148	0.363
AS (mm)	1.58	0.22	1.54	0.24	0.04	0.27	0.369	-0.209	0.195
PS (mm)	1.93	0.28	1.87	0.37	0.06	0.42	0.395	-0.247	0.124
Concentricity	0.20	0.07	0.18	0.07	0.02	0.09	0.285	0.099	0.542
**Dimension and position of condyle on axial plane**									
ML diameter (mm)	16.34	1.77	16.24	1.73	0.10	1.08	0.562	0.111	0.496
AP diameter (mm)	7.76	0.78	7.82	0.77	-0.05	0.46	0.456	-0.082	0.615
Axial angle (degree)	72.96	7.20	72.66	6.76	0.29	2.15	0.391	-0.069	0.673
Center distance to MSP (mm)	47.96	2.02	48.14	2.51	-0.18	1.55	0.473	-0.094	0.565
AP difference of condyle (mm)	-0.45	1.96	0.00	0.00	-0.45	1.96	0.160	-0.261	0.103
Vertical difference of condyle (mm)	-0.03	1.41	0.00	0.00	-0.03	1.41	0.885	0.011	0.945
**Mandibular dimension**									
Ramal height (mm)	46.18	3.55	46.73	3.69	-0.55	1.41	0.017*	-0.247	0.125
Mandibular body length (mm)	72.81	4.45	73.60	4.52	-0.79	2.02	0.018*	-0.339	0.032*
Total mandibular length (mm)	108.30	6.07	109.22	6.19	-0.91	1.55	0.001*	-0.328	0.039*
Gonial angle (degree)	126.01	4.39	126.24	4.76	-0.23	3.27	0.664	-0.287	0.072

SS, superior joint space; AS, anterior joint space; PS, posterior joint space; ML, mediolateral; AP, anteroposterior.

* P<0.05.

In the non-cleft group, no significant difference between the deviated and non-deviated sides was found in the superior, anterior or posterior joint space. The logPAe value was 0.020 on the deviated side and 0.018 on the non-deviated side, with no significant difference between the sides (P = 0.285).

Regarding the dimensions and positions of the condylar processes, no statistically significant differences were found between the deviated and non-deviated sides. In the evaluation of mandibular dimension, the ramal height, mandibular body length, and total mandibular length were significantly shorter on the deviated side than on the non-deviated side (P = 0.017, P = 0.018, and P = 0.001, respectively).

Table 5 shows the measurements of chin deviation in the UCLP and non-cleft groups.

**Table 5:** Comparison of chin deviation between the UCLP and non-cleft groups.

Chin deviation	UCLP group			Non-cleft group			P value
	Mean	SD	P value^†^	Mean	SD	P value^†^	
Value (mm)	1.63	1.33	0.001*	0.04	1.58	0.874	0.000*
Absolute value (mm)	1.82	1.04	-	1.32	0.86	-	0.037‡ *

Shapiro-Wilk test of normality;

^**†**^One sample t test;

^**‡**^Mann-Whitney U test (when variables were not normally distributed);

* P<0.05.

Of the 30 subjects with UCLP, 25 (accounting for 83.33%) showed chin deviation toward the cleft side. The average chin deviation was 1.63 mm (range -0.9 to 5.1 mm) toward the cleft side (P<0.001). Additionally, only 1 subject showed a chin deviation greater than 4 mm (5.1 mm) from the MSP to the cleft side, which was considered to be critical asymmetry. In the non-cleft group, the chins of 19 subjects deviated to the left side, and those of 21 subjects deviated to the right side. The average deviation was 0.04 mm (range -3.5 to 2.6 mm). The average absolute chin deviation in the UCLP group was 1.82 mm, which was significantly greater than the average absolute chin deviation (1.32 mm) observed in the non-cleft group (P = 0.037).

Pearson’s correlation analysis was used to examine the associations of chin deviation with the measurement differences between the two sides, and the results are shown in Tables 3 and 4.

In the UCLP group, the degree of chin deviation was positively correlated with the difference in the distance from the center of the condyle on each side to the MSP (r = 0.365, P = 0.047). Chin deviation was negatively correlated with the differences in ramal height (r = -0.386, P = 0.035), mandibular body length (r = -0.414, P = 0.023), and total mandibular length (r = -0.427, P = 0.018) between the cleft and non-cleft sides.

In the non-cleft group, the degree of chin deviation was negatively correlated with the differences in mandibular body length (r = -0.339, P = 0.032) and total mandibular length (r = -0.328, P = 0.039) on the deviated and non-deviated sides. However, no significant correlations were found between chin deviation and other measurements.

## Discussion

### 4.1 Subjects with class III skeletal relationship

It is widely accepted that subjects with CLP often present with varying degrees of class III skeletal deformities due to congenital defects and/or surgical disturbances. Previous comparative CLP studies enrolled non-cleft subjects with normal occlusion as the control groups, and comparisons were made between possibly class III CLP subjects and class I controls to investigate their facial asymmetries.

As previous studies have suggested, the rates of facial asymmetry in Caucasian and Mongoloid class III subjects were 40% and 11–25%, respectively, which were higher than those in class I and class II subjects [36, 37]. This difference might cause confusion regarding whether the difference in facial asymmetries between CLP and non-cleft subjects was due to cleft defects or to skeletal discrepancies. In stark contrast to previous studies, this study made comparisons between UCLP and non-cleft subjects with similar class III skeletal relationship to eliminate the underlying influence of different skeletal patterns on craniofacial asymmetry.

### 4.2 Three-dimensional imaging

Previous studies used conventional 2-D imaging, including panoramic, posteroanterior and oblique cephalometric radiographs, which was a projection of 3-D objects onto a 2-D surface; therefore, complete information concerning the craniofacial structures was not obtained. Furthermore, 2-D imaging studies were subject to error from the misidentification of landmarks, the overlap of anatomical structures, head posture changes, and magnification. To improve the validity and the reliability of imaging data in the current study, CBCT was used. Previous studies have suggested the advantages of CBCT in evaluating craniofacial asymmetry [24]. For instance, CBCT produces high-resolution images and presents the true morphology of the craniofacial skeleton because it does not suffer from projection- or distortion-related errors [38–40]. Additionally, the CT volume can be oriented by defined reference planes, and measurement accuracy is not affected by small variations in the patient’s head position [41]. In evaluation of the TMJ, CBCT images have also demonstrated greater validity and reliability than tomography and panoramic radiography [21–23].

### 4.3 Condylar-fossa relationships and bilateral symmetry

To investigate whether the condylar-fossa relationships were different between UCLP and non-cleft subjects with similar occlusion, the anterior, superior and posterior joint spaces were assessed on sagittal slices. For non-cleft subjects, most previous studies have suggested symmetric but noncentralized condyles in the temporomandibular fossa in normal occlusion and various malocclusions, and anterior joint spaces were significantly smaller than posterior joint spaces [42–44].

In the current study, instead of directly comparing the posterior and anterior spaces, we adopted the formula suggested by Pullinger to evaluate condylar concentricity [34]. According to Pullinger, an ideal expression evaluating joint concentricity should be responsive to joint size, and the direct difference between the anterior and posterior space can be affected by the varying sizes of joints. Since the expression of P/A is nonlinear, the expression of ${\text{log}}_{e}\frac{P}{A}$ was proposed. Condylar concentricity was defined as a range of ±0.25 on the ${\text{log}}_{e}\frac{P}{A}$ scale; thus, the condyle was considered to be anteriorly positioned when ${\text{log}}_{e}\frac{P}{A}$ was greater than 0.25 and to be posteriorly positioned when the result was less than -0.25, as described in Materials and methods.

In the non-cleft group, the average anterior space was smaller than the average posterior space, as previous studies have suggested. The ${\text{log}}_{e}\frac{P}{A}$ values for the deviated and non-deviated sides were 0.20 and 0.18, respectively. Forty subjects (100%) in the non-cleft group had either the right or left condyle centered in the fossa, and 27 subjects (67.5%) had both condyles centered.

To date, there has been a paucity of information on condylar-fossa relationships in CLP patients. In the UCLP group, the average ${\text{log}}_{e}\frac{P}{A}$ values for the cleft and non-cleft sides were 0.18 and 0.16, respectively. For all 30 subjects (100%) in the UCLP group, at least one condyle (the cleft or the non-cleft side) was centered in the fossa, and for 27 of the 30 subjects (90%), both condyles were centered. Therefore, the results indicated that the majority of subjects in both groups presented with centered condyles when evaluated using Pullinger’s formula.

In evaluating the bilateral symmetry of the condylar-fossa relationship, the value of ${\text{log}}_{e}\frac{P}{A}$ on the cleft side was 0.18, which was slightly greater than the value of 0.16 that was observed on the non-cleft side (P = 0.069). This result suggested that the condyle on the cleft side was slightly anteriorly positioned compared with the condyle on the non-cleft side. Admittedly, asymmetry might exist in the structures of the cranial base and the temporomandibular fossa, and the findings indicated functional adaptation of the condyle to the temporomandibular fossa, thus balancing the inferior growth of the mandible on the cleft side.

### 4.4 Dimensional and positional symmetry of the condyle

It is insufficient to evaluate the condylar symmetry of both sides only in the sagittal view. The axial plane is the most appropriate plane in which to assess the symmetry of the condyles in the AP and ML aspects, and it allows measurements of real condylar dimensions and angulations [43]. The axial plane was defined as the plane parallel to the FH plane and on which the geometric center of the condyle was located. Therefore, the axial planes of condyles on two sides might not have overlapped and would have needed to be measured separately.

#### 4.4.1 Diameter

In the current study, bilateral symmetry of the AP and ML diameters of the condyles were noted in both the UCLP and non-cleft groups, which suggested that neither deviations nor adaptive changes resulting from cleft defects were related to the condylar dimensions. For non-cleft subjects, the lack of asymmetry in these measurements is similar to observations reported in previous studies, in which the same methodology was applied for different types of malocclusions [43, 45].

#### 4.4.2 Position and rotation of the condyle

In the non-cleft group, no significant differences were found regarding the positions or rotations of the condyles between the deviated and non-deviated sides. In the UCLP group, the distance between the condyle and the MSP on the cleft side was significantly greater than that on the non-cleft side (P = 0.028), and the axial angle between the ML axis of the condyle and the MSP was significantly greater on the cleft side than on the non-cleft side (P = 0.001). These results indicated that the condyle on the cleft side was located at an increased distance from the MSP and was rotated outward at an increased axial angle.

According to previous studies, asymmetry in the position of the condyle, particularly on the ML aspect, is often associated with functional deviations due to occlusal characteristics. It is known that patients with UCLP often present with maxillary arch constriction and unilateral posterior crossbite on the cleft side. In addition, side preference during mastication is often associated with unilateral posterior crossbite features [46]. It is assumed that these functional and occlusal deviations might account for the positional and rotational asymmetries of the condyles in the UCLP group. This finding indicated that expansion of the upper arch and correction of the posterior crossbite might be helpful for relieving the asymmetry of the condyle, thus improving the lower facial symmetry. In addition, asymmetries of the craniofacial structures might have contributed to the results, and further studies are needed to evaluate the morphology and position of the cranial base and the temporomandibular fossa.

### 4.5 Mandibular dimensional symmetry

In the non-cleft group, the ramal height (Cp-Go), mandibular body length (Go-Me), and total mandibular length (Cs-Me) were significantly shorter on the deviated side than on the non-deviated side. These results were in accord with those of a previous study by Kwon et al [47]. Notably, however, the average differences between the two sides for these three measurements (-0.55 mm, -0.79 mm, and -0.91 mm, respectively) were all within the measurement error calculated using Dahlberg’s formula. Therefore, the results should be interpreted with caution and should be verified by further studies with more subjects.

In the UCLP group, the mandibular body length (Go-Me) and total mandibular length (Cs-Me) were shorter on the cleft side than on the non-cleft side. Kim et al reported similar results on mandibular length [27]. The gonial angle was significantly larger on the cleft side than on the non-cleft side, which is in agreement with the findings of Kurt et al [15]. However, the results differed from those of other previous studies. Veli et al [25] reported no significant differences in ramal height or mandibular length between the two sides in subjects with UCLP. By evaluating panoramic radiographs, Jena et al [48] reported significantly reduced ramal height and an increased gonial angle on the cleft side in UCLP subjects with near-normal maxillary growth but nearly symmetrical mandibles in UCLP subjects with severe maxillary hypoplasia.

These cited studies reflect confusion regarding the dimensional asymmetries of mandibles in subjects with UCLP. These inconsistencies among the results of the previous studies can be attributed to differences among the study subjects, the radiographs used, and the evaluation methods. Previous studies have enrolled UCLP subjects without addressing their skeletal and dental occlusal patterns, whereas in the current study, the subjects with presented with class III skeletal relationships. In addition, the average ages of the subjects with in previous studies were 16.6 years old [48] and 21.2 years old [25]. However, in the current study, the average age of the subjects with UCLP was 10.31 years old, and all of the subjects had mixed dentition before pubertal growth spurts. The subjects in the various age groups in these different studies represented different mandibular growth phases and showed diverse asymmetries.

### 4.6 Chin deviation

Because the mandibular chin is closely related to the perception of facial asymmetry, facial asymmetry was defined by the degree of chin deviation from the MSP in the current study. According to previous studies, a chin deviation of greater than 4 mm is regarded as clinically significant asymmetry [37, 49].

The average chin deviation was 1.63 mm to the cleft side, and 25 of 30 (83.3%) subjects showed deviation to the cleft side (P = 0.001). This result was consistent with that of a previous study by Kim et al [27], in which the average chin deviation was 1.59 mm to the cleft side, and 24 of 28 (85.7%) subjects showed deviation to the cleft side. Therefore, it was assumed that chin position tends to deviate to the cleft side in subjects with UCLP; however, only a few of these cases presented with clinically significant asymmetry.

The UCLP group showed an average absolute chin deviation of 1.82 mm, which was significantly greater than that observed in the non-cleft group (1.32 mm). The results indicated that both groups of subjects in the current study presented with mild lower facial asymmetry and that the asymmetries in skeletal class III UCLP subjects were more severe than those in non-cleft subjects with similar sagittal deformities.

### 4.7 Correlation analysis between each measurement and chin deviation

To investigate the factors contributing to chin deviation and the underlying reasons for the more severe asymmetry observed in subjects with UCLP, correlation analysis was performed between each measurement and chin deviation. In both groups, the results revealed that most of the factors that significantly differed between the two sides (mandibular dimensions in both groups, and distance from center of condyle to the MSP in the UCLP group) were associated with chin deviation. To be more specific, the differences in mandibular dimensional variables showed negative correlations with chin deviation, which was in accord with the results from previous studies in CLP and non-cleft subjects [27, 47]. This suggested that true mandibular dimensional asymmetries could be attributed to chin deviation in both groups.

Notably, the differences in the positions and angles between the condyles on each side and the MSP were positively correlated with chin deviation in the UCLP group (P = 0.027 and P = 0.052, respectively), whereas no significant correlations were found in the positional or rotational factors of the condyles in the non-cleft group. These results might indicate that functional factors, including positional and rotational changes, that existed in the UCLP group contributed to chin deviation in the UCLP group, whereas true mandibular asymmetries and not functional factors accounted for chin deviation in the non-cleft group.

It is also possible that the facial asymmetry was less significant in the non-cleft patient group than in the UCLP group; thus, the correlations between chin deviation and other measurements in the non-cleft patient group were not significant enough to be noted.

Also notable, the positional and rotational changes of the condyles did not compensate for the relatively shorter mandibular length on the cleft side, instead aggravating the chin deviation. This finding indicated an unsatisfactory compensatory mechanism in the subjects with UCLP, which might have resulted from discontinuous muscular structure, scar constriction, and surgical trauma, with the asymmetry possibly worsening as growth proceeds.

### 4.8 Future studies

Understanding the differences in the condylar-fossa relationship and in the mandibular asymmetry between UCLP and non-cleft subjects with skeletal class III malocclusion and identifying the correlation between mandibular asymmetry and chin deviation are of critical importance for the diagnosis and treatment planning of patients with UCLP.

In the current study, all of the subjects included had mixed dentition before pubertal growth spurts, and their facial asymmetries might not have been fully developed. Therefore, follow-up studies are needed to investigate whether asymmetry is related to growth and whether it is possible to predict the manner of mandibular growth.

## Conclusions

Our findings can be summarized as follows.

1. Regarding condylar-fossa relationships, 90% of UCLP and 67.5% of non-cleft subjects had both condyles centered according to Pullinger’s formula, and no significant correlation was found between the condylar-fossa relationship and chin deviation.
2. Chin position tended to deviate to the cleft side in the UCLP group, and the absolute value of chin deviation was significantly greater in the UCLP group than in the non-cleft group.
3. In assessing the rotation and position of the condyle, the axial angle and distance from the center of condyle to the MSP were significantly greater on the cleft side and were positively correlated with chin deviation in the UCLP group.
4. Considering the mandibular dimension, except for a larger gonial angle on the cleft side, the UCLP and non-cleft groups presented with consistent asymmetries, with a shorter mandibular body and total mandibular length on the cleft (deviated) side. The differences in mandibular body length and total mandibular length between the two sides were negatively correlated with chin deviation in both groups.
